# Fusion of External and Internal Prior Information for the Removal of Gaussian Noise in Images

**DOI:** 10.3390/jimaging6100103

**Published:** 2020-10-04

**Authors:** Ali S. Awad

**Affiliations:** Faculty of Engineering and Information Technology, Al-Azhar University, Gaza 79715, Palestine; aawad@alumni.stevens.edu; Tel.: +970-597197270

**Keywords:** noise reduction, external prior, internal prior, principal component analysis, denoise

## Abstract

In this paper, a new method for the removal of Gaussian noise based on two types of prior information is described. The first type of prior information is internal, based on the similarities between the pixels in the noisy image, and the other is external, based on the index or pixel location in the image. The proposed method focuses on leveraging these two types of prior information to obtain tangible results. To this end, very similar patches are collected from the noisy image. This is done by sorting the image pixels in ascending order and then placing them in consecutive rows in a new two-dimensional image. Henceforth, a principal component analysis is applied on the patch matrix to help remove the small noisy components. Since the restored pixels are similar or close in values to those in the clean image, it is preferable to arrange them using indices similar to those of the clean pixels. Simulation experiments show that outstanding results are achieved, compared to other known methods, either in terms of image visual quality or peak signal to noise ratio. Specifically, once the proper indices are used, the proposed method achieves PSNR value better than the other well-known methods by >1.5 dB in all the simulation experiments.

## 1. Introduction

Images can acquire noise during image acquisition, transmission, or recording. Gaussian noise is considered one of the most prevalent types of noise that may degrade an image and exists both, in wired and wireless channels [1,2]. Therefore, image denoising is a fundamental process that should be implemented before any advance image processing tasks, and remains challenging. A wide range of algorithms have been proposed in the literature using prior information for estimating the noisy images. Some approaches use the input noisy image as a supplement or as prior information, such as NLM [3], BM3D [4], PGPCA [5], LPG-PCA [6], and WNNM [7]. Other approaches use external images as prior information, such as EPLL [8], dictionary-based denoising methods [9] and others [10,11]. To better estimate the noisy patches, other approaches use a combination of internal and external prior information [12,13]. The authors in [14] propose a new method called expectation-maximization adaptation to adapt the external database using the internal one and to decrease the amount of training data. In [15] the authors proposed k-nearest neighbor-based collaborative filtering in which a query of similar patches is recommended, with the help of the similar patches in an internal or external database. Other methods based on wavelet shrinkage methods [16,17,18,19] are proposed and many others are used to build complex mapping functions between a corrupted and a clean version of an image, as described in [20,21]. The low-rank models, described in [22,23,24], are used for image restoration in order to deliver favorable results. Moreover, different methods based on deep learning have been explored for image denoising as explained in [25,26,27,28]. 

In this paper, two types of prior information are used; one is external, based on the indices of the training image, and the other is internal, based on the similarity between the pixels in the overlapped patches. Extensive simulation experiments are implemented on different images to illustrate that the proposed method delivers outstanding results, either in terms of visual image quality based on human perception or peak signal to noise ratio (PSNR). Note that sometimes PSNR provides insufficient description about the restored image, therefore human visual perception is essential. This paper is outlined as follows: In the first section an introduction is presented. Then, in Section 2 and Section 3 the algorithm description and the simulation results are given. Finally, a conclusion is outlined in Section 4.

## 2. Algorithm Description 

**Figure 1:**
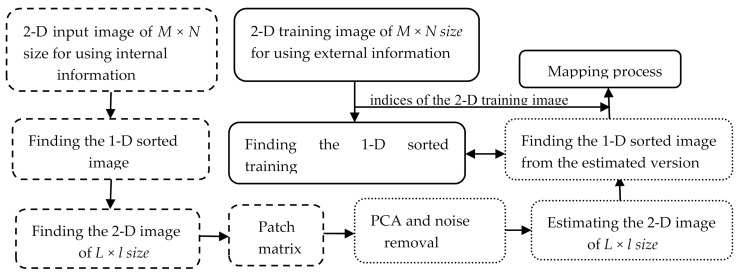
Block diagram describing the steps of the proposed algorithm in denoising corrupted images.

In this paper, a patch-based approach is proposed, in which two types of prior information are used to help estimate the noisy patches. The first type is similarity-based and the second is index-based. The first type is based on constructing very similar patches. They are constructed by ordering all the pixels in the noisy image in ascending fashion in a one-directional (1-D) image. Then, the pixels in the 1-D version are separated in rows in a two-directional (2-D) image, where each row includes a specific number of pixels. The number of columns in the 2-D image is usually less than the number of rows. Then, a sliding window moves over the entire new image to produce a patch matrix. After using a principal component analysis (PCA) on the patch matrix, the second type of prior information is used. In other words, the question is how we can rearrange the pixels in the estimated patch matrix based on their new values so that the output image achieves the optimum result. Since the new estimated pixels have similar values to those of the original ones, the optimum solution is to relocate each estimated pixel in the index or location of its equivalent in the original image. If the original indices are not saved in a library, then the next best solution is to use the indices of the image that are estimated by another efficient method, particularly those estimated from a low-noise-corrupted image. In any case, it is recommended that a library including the indices of a large number of images be established or that research to estimate the image indices be conducted. Another important point is that the proposed algorithm is self-terminating. In other words, it is terminated once the root-mean-square error (RMSE) between the restored and corrupted versions reaches a minimum. Figure 1 shows the denoising steps of the proposed algorithm, starting from converting the noisy image to a 1-D image, then applying PCA on the patch matrix until the relocation process in which the estimated pixels are placed in a 2-D image based on the indices of the corresponding pixels in the training image. The algorithm steps are described in detail as shown below:

### 2.1. Input Image for Using Internal Information 

Consider a 2-D image *X* of *M* × *N* size corrupted by independent and identically distributed Gaussian noise *ε* of zero mean and variance *σ*^2^ as *ε~N*(0, *σ*^2^). Mathematically, *x* = *y* + *ε*, where *x*, *y* ∈ ℝ*^M^*×*^N^*, *y* is the cleaned pixel and *x* is the corrupted pixel. Define the corrupted image as *X*:

$$X=\left[.\begin{array}{ccccccc} x_{11} & x_{12} & . & . & . & . & x_{1N}\\ . & x_{22} & . & . & . & & \\ . & . & x_{33} & . & . & & \\ . & & & . & & & \\ x_{M1} & & & & & & x_{MN} \end{array}\right]$$

### 2.2. Finding the 1-D Sorted Image

The first problem is how we can achieve maximum similarity or minimum intensity distance between each two consecutive pixels. To this end, assume a pixel *x_i’j’_* in location *i’*, *j’* in the corrupted image. Then, we have to search all the other locations *ij*, *i* = 1, 2, …, *M*, *i ≠ i’*, *j* = 1, 2, …, *N*, *j ≠ j’*, in the image in order to find the pixel that provides the maximum similarity *S* or minimum intensity distance with pixel *x_i’j’_* as:

(1) $$S=\underset{ij}{\arg\min}\;\;\left|x_{i’j’}-x_{ij}\right|$$

Pixels *x_ij_* and *x_i’j’_* are expected to be two consecutive pixels in a new image. The suggested solution for the problem mentioned in step 2 is to order the pixels in the image *X* in an ascending fashion in a one-dimensional 1-D vector $\bar{X}$ to maintain maximum similarity or lowest intensity difference between each two consecutive pixels, which are defined as the pixels that follow each other in the spatial domain as:

(2) $$\bar{X}=\{{\bar{x}}_{1,j}\;\;|\;\;{\bar{x}}_{1,j}\leq {\bar{x}}_{1,j+1},\;\;j=1,2,\ldots ,(MN-1)\}$$

### 2.3. Finding the 2-D Image of Lxl Size and Patch Matrix

Reshape matrix $\bar{X}$ to be with similar consecutive rows *r*’s where number of rows equal to *L* = *M* × *a*, a is an integer number. Each row *r* has a length of ℓ=N/a pixels. As a result, a matrix *R* of L×ℓ size is obtained. It helps in finding patches of similar elements:

(3) $$r_{1}=\{{\bar{x}}_{1},{\bar{x}}_{2},{\bar{x}}_{3},\ldots {\bar{x}}_{l}\}\;r_{2}=\{{\bar{x}}_{1+\ell },{\bar{x}}_{2+\ell },{\bar{x}}_{3+\ell },\ldots {\bar{x}}_{2\ell }\},\ldots ,\;\;r_{L}=\{{\bar{x}}_{(L-1)\ell +1},{\bar{x}}_{(L-1)\ell +2},{\bar{x}}_{(L-1)\ell +3},\ldots {\bar{x}}_{L\ell }\}\;r_{k}=\{{\bar{x}}_{1,j+(k-1)\ell }\;|j=1,2,\ldots ,\ell ;k=1,2,\ldots ,L\}\;R={\left[r_{1},r_{2},r_{3},\ldots ,r_{L}\right]}^{T}$$

The aim of the patch matrix *P* is to increase the redundancy of each pixel in the local region. To this end, a sliding window of *w × w* size is moved over the image *R* and then inserted as a column vector in *P*. Thus, the size of *P* is equal to *w^2^ × MN*. Note that matrix *R* is padded in all directions by (*w* − 1)/2 rows and (*w* − 1)/2 columns which are mirror reflections of the rows and columns along the border. 

### 2.4. PCA and Noise Removal

To remove the noise from the patch matrix *P* the covariance matrix *Σ* of *P* is calculated as,

(4) $$\sum =E[(P-\mu I)(P-\mu I{)}^{T}]=E[CC^{T}]$$

where *C = P − µI*, *µ* is a column vector that includes the mean of all the elements *p’s* in each row in the patch matrix *P*, i.e., $\mu =\sum_{i=1}^{i=w^{2}}\sum_{j=1}^{j=MN}p_{ij}$ and *I* is a unity row vector of size 1 × *MN*. More specifically, the centralized matrix *C* is attained if each element in patch *P* is decreased by the mean *µ* of the element’s row. Since *Σ* is symmetric, it is valid to use Eigenvalue decomposition by which *Σ = U Λ U^T^*, where *U* and *Λ* are Eigenvector, and eigenvalue matrices, respectively. 

Then, we have to find the estimated centralized matrix $\hat{C}$ that achieves minimum error *E* with C as:

(5) $$E=\underset{Th}{\arg\min}|\hat{C}-C|$$

The projection of *C* on *U* is defined by matrix *B* and the projection of $\hat{C}$ on *U* is defined by matrix $\hat{B}$ as:

(6) $$Β=CU\;\mathrm{and}\;\hat{B}=\hat{C}U$$

To estimate $\hat{C}$, any small components *p*, *p* *∈* *Β* are neglected based on a predefined threshold *Th*, i.e., *p =* 0 *if (p/p_max_) < Th*, *p_max_* is the maximum value in *Β*. The result is a new matrix $\hat{B}$ that includes the remaining informative components. Therefore, $\hat{C}=\hat{B}\;\;U^{T}$. Note that *U^T^ = U*^−1^ because matrix *U* is an orthogonal matrix. Finally, the estimated patch matrix $\hat{P}$ based on Equations (4) and (6) are obtained as:

(7) $$\hat{P}=\hat{B}\;\;U^{T}+\mu I$$

### 2.5. Finding the Estimated Lxl Size Image and Its 1-D Sorted Image

Matrix $\hat{P}$ is aggregated in a way opposite to that mentioned in step 4. The result is an estimated version $\hat{R}$ of *L ×* ℓ size. Then, after removing the padded rows and columns, is ordered in an ascending fashion to create a 1-D estimated version $\hat{\bar{X}}$ of matrix $\bar{X}$ and is defined as: $\hat{\bar{X}}=[{\hat{\bar{x}}}_{1,\ldots ,},{\hat{\bar{x}}}_{2,\ldots ,}{\hat{\bar{x}}}_{MN}]$.

### 2.6. Finding the Indices of the 2-D Training Image as an External Information

2-D training image $I_{2}^{t}$ and its 1-D ascending ordered version $I_{2}^{t}$ are used in this step. Each pixel in $I_{1}^{t}$ vector has a corresponding index (*a_i_,a_j_*) in the $I_{2}^{t}$ version. Indices *(a_i_,a_j_)* are saved in a new index image *I_index_* which is defined with matrix $\hat{\bar{X}}$ as:

(8) $$I_{index}=\;\;[(a_{1},a_{2}),(a_{3},a_{4}),\ldots .,(a_{M},a_{N})]$$

(9) $$\hat{\bar{X}}=\{\hat{\bar{x}}|{\hat{\bar{x}}}_{1,j}\leq {\hat{\bar{x}}}_{1,j+1},j=1,2,\ldots ,(MN-1)\}$$

### 2.7. Mapping Process

If matrices $\hat{\bar{X}}$ and $I_{1}^{t}$ have similar pixels, then they should have the same indices mentioned in matrix *I_index_*. Thus, a mapping or a relocation process is performed to locate each pixel in to a new location in $\hat{X}$. The new location for each pixel in $\hat{\bar{X}}$ is specified by its corresponding index described in *I_index_* as follows:

(10) $$\hat{X}(I_{index})=\hat{\bar{X}}\;{\hat{\bar{x}}}_{1}\to (a_{1},a_{2}),\;{\hat{\bar{x}}}_{2}\to (a_{3},a_{4}),\;\ldots ,{\hat{\bar{x}}}_{MN}\to (a_{M},a_{N})$$

Note that indices that are similar to those of the original pixels always deliver the best results. However, indices of a restored version from a low-noise-corrupted image will provide superior results as well. If these indices are not available, one may create a library that includes the indices of the best-known images, or may one conduct research to build a model for prediction of indices using deep learning techniques. 

### 2.8. Algorithm Termination

To terminate the algorithm, the minimum RMSE between the restored image and the corrupted version should be calculated, and it is important to mention that minimum RMSE is achieved at a certain threshold value as follows:

(11) $$\underset{Th}{\arg\max}\left\{PSNR\right\}=\underset{Th}{\arg\min}{\left\{\frac{\sum_{i}^{M}\sum_{j}^{N}{\left(x_{ij}-{\overset{⌢}{x}}_{ij}\right)}^{2}}{MN}\right\}}^{1/2}$$

## 3. Simulation Results

The purpose of this section is to illustrate the performance of the proposed method in comparison with state-of-the-art methods such as EPLL [8], BM3D [4], and PGPCA [5]. Each method is evaluated objectively, based on PSNR, and subjectively based on the visual quality of the restored images. The proposed method utilizes two types of prior information to restore the corrupted image. The first is taken from similar pixels of similar objects in the image. The second is taken by using the indices of the original image or the indices of other images having pixel values similar to the original values. Therefore, the implementation of the proposed method becomes very easy once a database including indices of many images is collected, particularly the indices of images that are used the most. One may conduct research to estimate the proper indices for the estimated versions. In the current study, 8- bit gray level images having an intensity range from 0 to 255 and of 512 × 512 size are used in all the simulation experiments. Each image is converted first to a 1-D version ordered in ascending fashion. Then, to construct a 2-D image of similar rows, the 1-D version is converted to a new matrix of *L =* 512 *×* 16 rows and ℓ *=* 512/16 columns. The window used in the simulation experiments is of the size *w × w =* 11 *×* 11, which is then inserted in the patch matrix as a column of size {(11 × 11),1}. To select the optimum threshold value *Th* for each experiment, the algorithm is executed seven times from *Th* = 0.1 to *Th* = 0.7. Then, the threshold which provides the optimum result is selected. Zero mean Additive White Gaussian Noise, AWGN, is generated using the MATLAB (version R2014a) randn fuction and added to the image in different amounts based on the standard deviation σ. The advantage of the proposed method is that two of its parameters remain unchangeable, but the other is changed or tuned for optimum results. Note that the number of columns *l* of the new converted image is chosen to be smaller than the main image columns, i.e., *l < N*, in order to divide the image into similar objects with similar rows. Another main advantage of the proposed method is that the algorithm is self-terminated at a certain threshold value which achieves minimum root-mean-square error between the restored and corrupted versions. To study the effect of each parameter on the restoration performance, several tables and figures are proposed. 

Table 1 shows the restoration performance in terms of PSNR when the corrupted image is reshaped at different sizes, each having a decreased number of columns *l* and an additional number of rows *L*. It is notable from the table that as the number of columns decreases gradually, but to a specific limit, the restoration performance increases gradually. In other words, one can say that at *l* = 128, 64, and 32 the proposed method provides satisfactory results, but for small image widths, i.e., *l* = 16, poor results are obtained. Note that the values of the other parameters *w × w* and *th* value remain constant. Thus, it is better to decrease the number of columns in the corrupted image to divide the image into objects of similar rows.

Table 2 shows the restoration performance in terms of PSNR at different window sizes. One can observe that as the size of the window *w* × *w* increases, but to a specific size, the restoration performance increases. In other words, sizes of *w × w* = 9 × 9*,* and 11 × 11 provide satisfactory results. One can conclude that within these sizes, we may find objects of similar rows. The threshold value *Th* and the new size of the corrupted image *L* × *l* remain intact. The abbreviation Lena(original) denotes that the indices of the restored image are obtained from the original image; but the notation Lena(BM3D,5) in the table denotes that the indices of the restored image are obtained from the restored version that was achieved due to restoring a corrupted Lena image at *σ* = 5 by the BM3D method. 

**Figure 2:**
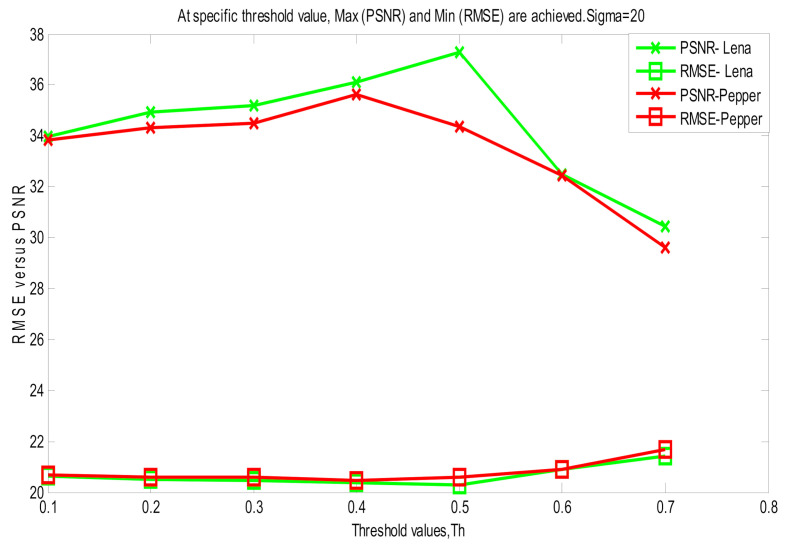
Comparison between RMSE and PSNR at different threshold values for different images. Once the threshold value achieves minimum RMSE between the restored and the corrupted versions, the algorithm terminated.

Figure 2 illustrates the threshold effect in restoring Lena and Pepper images corrupted at *σ* = 20. It is obvious that for each image there is a certain threshold that provides maximum PSNR and minimum root-mean-square error RMSE between the restored and the corrupted versions. Note that if RMSE reaches a minimum value, the algorithm is terminated automatically.

Table 3 illustrates the restoration performance of the proposed method at different threshold values and at *σ* = 30. The other parameters remain unchanged. It is clear from the table that at a certain threshold value, minimum RMSE or maximum PSNR is obtained.

At this threshold value the algorithm is self-terminated. Note that in all the simulation experiments one iteration is used, as the optimum parameters are used in this iteration.

Table 4 demonstrates the consumed time in seconds for different methods in restoring two corrupted versions at *σ =* 20*,* one for the Lena image and the other for the Pepper image. It is obvious that the proposed method is fast and provides computational complexity lower than other methods. It is clear that the BM3D method is the fastest one. However, the proposed method delivers the best restoration performance. These results are implemented in MATLAB version R2014a on a computer with an HP ENVY TS 14 Sleekbook Intel Core i5-4200U@1.60 GHz CPU. 

**Figure 3:**
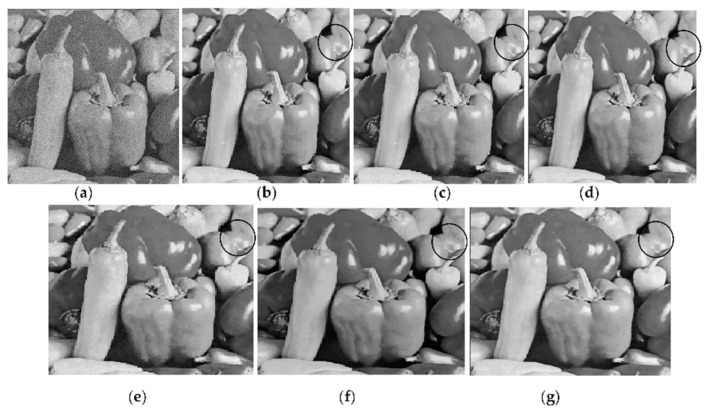
Outputs of the proposed and other methods in restoring Pepper image corrupted at σ = 20. In proposed method, each output uses a different indices: (**a**) Corrupted image; (**b**) new with indices of original image, PSNR = 35.62; (**c**) new with indices of BM3D, PSNR = 34.06; (**d**) New with indices of PGPCA, PSNR = 34; (**e**) New with indices of EPLL, PSNR = 31.27; (**f**) output from BM3D, PSNR = 33.64; (**g**) output from PGPCA, PSNR = 32.59.

Figure 3 shows four versions restored by the proposed method at *Th =* 0.4 from a corrupted Pepper image at *σ* = 20. Each version uses different indices. Three different indices are obtained from three different versions restored by EPLL, PGPCA and BM3D methods. Two versions are obtained by PGPCA and BM3D as a result of restoring corrupted images at *σ* = 5. The other version is restored by EPLL from the corrupted version at 𝜎 = 20. 

**Figure 4:**
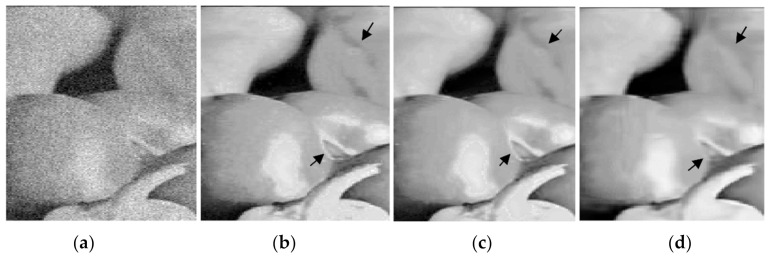
Enlarged parts from the outputs b, c, and f mentioned in Figure 3 to show the restoration performance of the proposed and BM3D methods in restoring Pepper image corrupted at *σ* = 20: (**a**) Part from the corrupted Pepper image; (**b**) New with indices of original image; (**c**) New with indices of BM3D(*σ* = 5); (**d**) Output from BM3D.

It is clear that the versions using the indices of the original image in (b) and the indices of the restored version obtained from low-noise-corrupted image at *σ* = 5 in (c) and (d) deliver superior results that are better than BM3D and PGPCA, either in terms of PSNR or visual image quality. One can note that the indices of PGPCA are used in (d) for delivering better a PSNR value than that obtained by the BM3D method in (f), although BM3D is one of the best methods in this field. Figure 4 includes enlarged parts attained from the restored images shown in Figure 3a,c,f. Black circles in Figure 3 or black arrows in Figure 4 clearly indicate that the images restored by BM3D and PGPCA have less detail than the others. 

**Figure 5:**
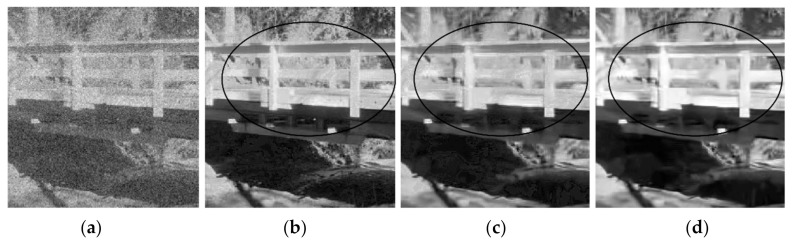
Enlarged parts from the restored Bridge images attained by the proposed and BM3D methods to show the restoration performance of each in restoring Bridge image corrupted at *σ* = 30: (**a**) Part from the corrupted Brige image; (**b**) New with indices of restored version by BM3D from low-noise-corrupted image at *σ* = 5; (**c**) New with indices of restored version by BM3D from a corrupted image at *σ* = 30; (**d**) Output from BM3D.

Figure 5 shows an enlarged part from the bridge image from a version corrupted at *σ* = 30 restored by different methods. *Th =* 0.4 is used in the proposed method. It is clear that the new method with the indices of the restored version obtained from low-noise-corrupted image at *σ* = 5, restored by BM3D, in (b) provides an image with more detail and a more pleasing appearance than the others as indicated in the surrouded area in each restored version. 

**Figure 6:**
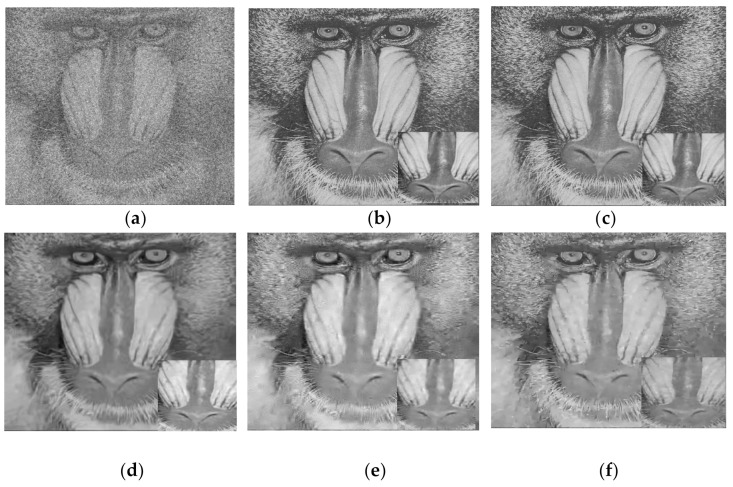
Outputs of the proposed algorithms compared with different methods in restoring Baboon image corrupted at *σ* = 50: (**a**) Corrupted image; (**b**) (New, indices of original, *σ* = 0, PSNR = 24.27); (**c**) (New, indices of BM3D, *σ* = 5, PSNR = 23.91); (**d**) BM3D, PSNR = 22.28; (**e**) PGPCA, PSNR = 21.97, (**f**) EPLL, PSNR = 22.39.

Figure 6. Shows the performance of different methods in restoring the Baboon image corrupted at *σ* = 50. In this figure, the following notation is used (new, b, c, d) where “new” denotes the new method; b: denotes an image or name of a method, which means that the new method uses the indices of the image in b or the indices of a restored image attained by the method mentioned in b; c: denotes that the restored image in b is obtained from a version corrupted at *σ* = c; d: denotes the performance of the proposed method in terms of d = PSNR. It is clear that the proposed algorithm, using threshold *Th = 0.5*, delivers superior results with the indices attained from the original image and the restored version produced from a low-noise-corrupted image at *σ* = 5. It is evident that the results of the new method are better than those of any of the other methods.

Table 5 and Table 6 illustrate the restoration results in terms of PSNR for different methods in restoring different images at *σ* = 20, and *σ* = 30, respectively. It is clear that the new method with the indices of original or low noise restored version by PGPCA or BM3D at *σ* = 5 delivers the best results.

## 4. Conclusions

A new method for the removal of Gaussian noise is explored in this paper. The proposed method is based on internal and external prior information used in estimating the corrupted pixels. The first type of information is achieved by gathering the most similar patches from the noisy image. The second is utilized when the restored pixels are relocated in new positions in the image. Since the restored pixels have new values similar to those of the original version, it is preferable to relocate them with the indices of the original image or with the indices of a restored version, similar to the original image. Therefore, establishing a library that includes the indices of as many different images as possible will be helpful and should be considered in future work. Finally, the algorithm is self-terminating once the root-mean-square error between the estimated and corrupted image reaches a minimum. Simulation experiments proved that the new method outperforms the other well-known methods and yields extraordinary results.

## Figures and Tables

**Table 1:** The effect of resizing the corrupted image in terms of PSNR. *Th* = 0.5(lena), *Th* = 0.4(pepper), *Th* = 0.4(bridge), indices from original image, and *w × w =* 11 × 11.

*σ* = 20	512 × 512	1024 × 256	2048 × 128	4096 × 64	8192 × 32	16,384 × 16
Lena	33.36	33.92	35.12	36.86	37.2	28.26
Pepper	33.7	34.07	34.35	36.75	35.62	30.88
Bridge	36.05	36.38	36.80	37.20	34.44	28.32

**Table 2:** The effect of changing the window size *w* × *w* in terms of PSNR. *Th* = 0.5(Lena), *Th* = 0.4(pepper),*Th* = 0.4(bridge), *Th* = 0.6(Lake).

*σ* = 20	7 × 7	9 × 9	11 × 11	13 × 13	15 × 15	17 × 17
Lena(original)	35.44	35.4	37.2	36.54	36.12	34.00
Lena(BM3D,5)	34.07	34.05	35.31	34.92	34.62	32.98
Pepper(original)	36.21	36.63	35.62	33.42	33.46	33.72
Bridge(original)	35.02	34.51	34.44	33.34	33.25	32.54
Lake(original)	33.87	37.19	35.41	31.64	31.34	32.02

**Table 3:** The effect of changing the threshold value in terms of RMSE and PSNR (RMSE/PSNR). At minimum RMSE or maximum PSNR the algorithim terminated.

*σ* = 30	*Th* = 0.1	*Th* = 0.2	*Th* = 0.3	*Th* = 0.4	*Th* = 0.5	*Th* = 0.6
Lena	31.52/28.3	31.24/29.1	31.17/29.4	30.63/32	30.46/33.3	30.79/31
Pepper	31.43/28.6	31.25/29.2	31.15/29.6	30.75/31.4	30.41/34	30.82/31
Bridge	31.06/29.94	31.02/30.14	30.79/31.18	30.5/33.17	30.7/31.76	31.35/28.88
Baboon	31.57/28.3	31.4/28.8	31.17/29.5	30.550/32.9	30.554/32.76	30.80/31.12
Lake	32.55/26.1	32.27/26.6	32.15/26.9	32/27.2	30.53/33	30.50/33.2

**Table 4:** Comparison between different methods in terms of time consumption in seconds.

*σ* = 20	New(original)	New, BM3D(5)	BM3D	PGPCA	EPLL
Lena	20.20	20.49	8.43	16.76	821.04
Pepper	20.96	20.42	8.96	17.51	870.67

*σ* = 20

New(original)

New, BM3D(5)

BM3D

PGPCA

EPLL

**Table 5:** Comparison between different methods in restoring different images at σ = 20.

*σ* = 20	Original, New	PGPCA(5), New	BM3D	PGPCA	EPLL
Lena(0.5)	37.2	35.24	33.29	32.45	32.9
Pepper(0.4)	35.62	34.06	33.64	32.59	33.29
Lake(0.6)	35.41	32.92	30.33	30	30.39
Boat(0.3)	33.08	31.79	31.12	30.39	30.96
Baboon(0.4)	37.68	33.57	26.57	26.23	26.73
Fruits(0.3)	36.80	34.64	32.76	31.70	32.67
Cat(0.3)	36.93	34.22	29.85	29.55	29.65

**Table 6:** Comparison between different methods in restoring different images at σ = 30.

*σ* = 30	Original, New	BM3D(5), New	BM3D(10), New	BM3D	PGPCA
Lena(0.5)	33.3	32.36	31.44	31.5	31.29
Pepper(0.5)	34	32.91	31.99	31.94	31.46
Bridge(0.4)	33.17	31.34	29.01	25.43	25.92
Baboon(0.4)	32.9	30.96	28.48	24.52	25
Lake(0.6)	33.2	31.36	29.71	28.53	28.87
